# Geranylgeraniol and Neurological Impairment: Involvement of Apoptosis and Mitochondrial Morphology

**DOI:** 10.3390/ijms17030365

**Published:** 2016-03-11

**Authors:** Annalisa Marcuzzi, Elisa Piscianz, Marina Zweyer, Roberta Bortul, Claudia Loganes, Martina Girardelli, Gabriele Baj, Lorenzo Monasta, Claudio Celeghini

**Affiliations:** 1 Piazzale Europa 1, University of Trieste, Trieste 34127, Italy; zweyer@units.it (M.Z.); bortul@univ.trieste.it (R.B.); claudia.loganes@gmail.com (C.L.); gbaj@units.it (G.B.); cceleghini@units.it (C.C.); 2 Institute for Maternal and Child Health—IRCCS “Burlo Garofolo”, Via dell’Istria, 65/1, Trieste 34137, Italy; elisa.piscianz@gmail.com (E.P.); martina.girardelli@burlo.trieste.it (M.G.); lorenzo.monasta@burlo.trieste.it (L.M.)

**Keywords:** cholesterol pathway, apoptosis, neuroinflammation, mevalonate, mitochondria, neuronal death

## Abstract

Deregulation of the cholesterol pathway is an anomaly observed in human diseases, many of which have in common neurological involvement and unknown pathogenesis. In this study we have used Mevalonate Kinase Deficiency (MKD) as a disease-model in order to investigate the link between the deregulation of the mevalonate pathway and the consequent neurodegeneration. The blocking of the mevalonate pathway in a neuronal cell line (Daoy), using statins or mevalonate, induced an increase in the expression of the inflammasome gene (*NLRP3*) and programmed cell death related to mitochondrial dysfunction. The morphology of the mitochondria changed, clearly showing the damage induced by oxidative stress and the decreased membrane potential associated with the alterations of the mitochondrial function. The co-administration of geranylgeraniol (GGOH) reduced the inflammatory marker and the damage of the mitochondria, maintaining its shape and components. Our data allow us to speculate about the mechanism by which isoprenoids are able to rescue the inflammatory marker in neuronal cells, independently from the block of the mevalonate pathway, and about the fact that cell death is mitochondria-related.

## 1. Introduction

**Figure 1:**
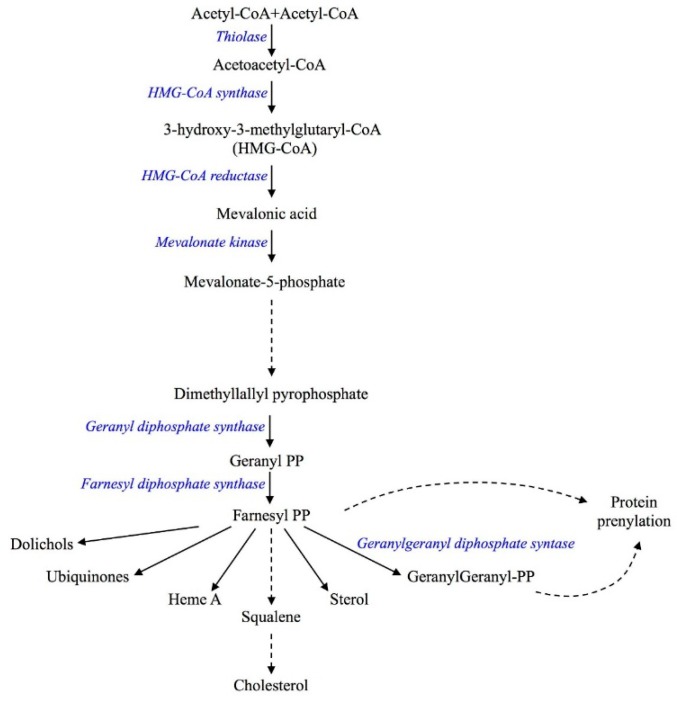
Schematic representation of the mevalonate pathway. The solid arrows represent the single enzymatic reaction while the dashed arrows indicate multiple reaction steps.

The cholesterol pathway, commonly called the mevalonate pathway, is a crucial biochemical process present in all higher eukaryotes and many bacteria [1,2]. It is a fundamental step for the biosynthesis of several molecules involved in multiple processes as diverse as terpenoid synthesis, protein prenylation, cell membrane maintenance, hormones synthesis, protein anchoring and *N*-glycosylation [3,4] (Figure 1).

The inborn deregulation of the cholesterol pathway can lead to various pathological consequences. Several of these diseases show a genetic mutation of various compounds along the mevalonate pathway, such as coenzyme q10 deficiency (OMIM #612016; CoQ10) and mevalonate kinase deficiency (OMIM #260920; MKD) [5,6].

Most of these diseases are rare and neglected, and are characterized by severe neurological involvement that represents a convergent consequence of pathogenetic mechanisms linked to the deregulation of the cholesterol pathway [7].

Our group studied MKD in cellular and animal models—In which the genetic defect of human MKD is mimicked by the biochemical blockade of the mevalonate pathway—Obtained using several compounds able to affect the pathway at different levels, such as statins that block the mevalonate pathway on enzyme HMG-CoA reductase [8] (Figure 1).

In addition to their lipid-lowering effect, recent data have shown that statins play a neurotoxicity role in cells by inhibiting proliferation, and inducing programmed cell death.

Structural differences of various statins (Atorvastatin, Fluvastatin, Lovastatin, Pitavastatin, Pravastatin, Rosuvastatin, Simvastatin) support different pharmacokinetic responses, and this is particularly evident in the diseases of the nervous system. It is thus essential to emphasize the importance of identifying the precise mechanistic differences between statins in order to make the best therapeutic decision, even though some molecular mechanisms induced by the statins have not yet been fully understood [9].

To date it is commonly assumed that the biosynthesis of cholesterol in the central nervous system occurs *in situ*, as a closed system, not dependent on systemic circulation [10,11,12].

The aim of our study is to understand the neurological effects of the cholesterol pathway blockade. An MKD-neuronal model *in vitro* represents an effective tool to investigate the mechanism of activation of inflammation linked to abnormalities in cholesterol traffic.

Recently we have demonstrated that the block of the cholesterol pathway on a neuronal cell line induces caspase 3- and caspase 9-dependent programmed cell death [13,14,15].

To date, it is still unclear if the neuroinflammation induced by the block of the mevalonate pathway depends on the accumulation of mevalonate or on the inhibition/block of the biochemical process, and if both processes are associated to inflammation triggered by the mitochondria.

Several recent literature reports have drawn attention into the role of mevalonate-derived isoprenoids, such as GGOH, in the regulation of several cellular processes and in the control of inflammatory response to enzymatic regulation and response to oxidative stress [16,17]. Notably, environmental factors can act in different ways in this scenario: nutritional factors can influence the availability of substrates and the regulation of different enzymes in the mevalonate pathway; immune stimuli such as infections and vaccinations can trigger an inflammatory response that involves several prenylated proteins [18]; oxidative stress of various natures can exceed the antioxidant capacity of a defective mevalonate pathway, as described by Campia *et al.*, that proved that GGOH co-treatment with statins was able to rescue toxicity in THP-1 cells [19].

Finally, it can be interesting to understand if mechanisms of rescue, such as deployment of isoprenoids, are able to restore the inflammatory mechanisms even in the case of accumulation of mevalonate, as occurs when the pathogenic processes are induced by the block of the pathway [20].

To establish the comparable inflammatory effects on the different inhibition of the mevalonate pathway due to the accumulation of the mevalonate or limiting the biosynthesis of compounds along this biochemical process is fundamental to study the different levels of severity. The different approach in the study of the pathogenesis of the mild and severe forms of MKD, should take into account this molecular aspect: the reinforcement of the thesis according to which the MKD-inflammatory phenotype is due to lack of isoprenoids, independently from the cause in the central nervous system, would be crucial to identify an efficient pharmacological strategy.

For this reason, it is important to understand the role of the hypothetical accumulation of mevalonate or of the lack of mevalonate-derived isoprenoids, in order to shed light on some steps of the molecular pathogenesis of several diseases involved in the deregulation of the cholesterol pathway.

## 2. Results

### 2.1. Programmed Cell Death Triggered to Deregulation of the Mevalonate Pathway

**Figure 2:**
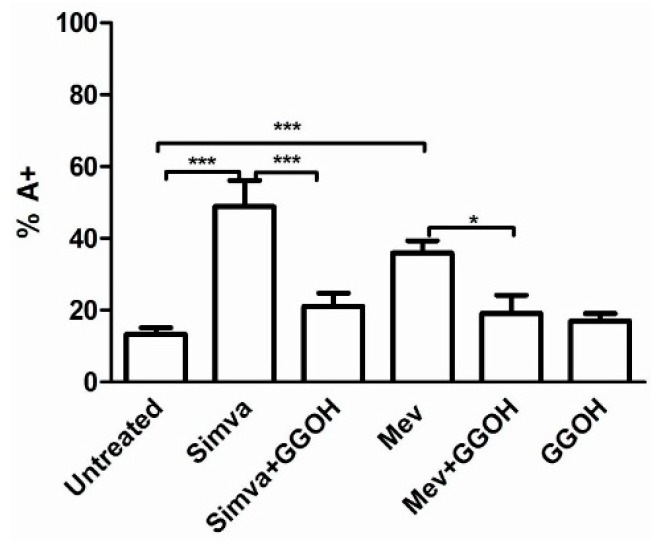
Daoy cell line was incubated with Mevalonate (10 mM) and/or Simvastatin (10 μM) for 24 h and with GGOH (50 μM) for an additional 24 h. Programmed Cell Death is expressed as percentage of Annexin V positive cells (%A+) and bars represent the means % ± SD of five independent experiments. Data analyses were performed with one-way ANOVA followed by Bonferroni post-test; * *p* < 0.05; ** *p* < 0.01, *** *p* < 0.001.

The Apoptosis rate of Daoy treated with different compounds was analysed with Annexin V-FITC Apoptosis Detection Kit. The treatment of cells with Simvastatin and Mevalonate showed a statistically significant increase in the number of apoptotic cells, with a percentage of Annexin V-positive cells respectively of 48.86% and 35.95%, compared to the 13.39% of the untreated cells (** *p* < 0.01; *** *p* < 0.001) (Figure 2). Our results prove that Simvastatin is more effective and quick in blocking the mevalonate pathway and inducing cell death in comparison to mevalonate.

Treating cells with GGOH alone did not modify significantly the cell death rate (16.95%), while administration in combination with Simvastatin or Mevalonate, restored the basal condition, nullifying the alteration induced by Simvastatin and Mevalonate (from 48.86% to 21.17% for Simvastatin, *** *p* < 0.001; from 35.95% to 19.13% for Mevalonate, * *p* < 0.05). The co-treatment of GGOH with Statin and Mevalonate decreased partially the damage induced by this latest compounds and enhanced the resistance of our neuronal cells to this disorder (Supplementary Figure S1).

### 2.2. Mitochondrial Dysfunction Linked to Block of Cholesterol Production

To evaluate mitochondrial damage after the block of the mevalonate pathway in the Daoy cell line, we assessed the impact of statins and mevalonate treatments on the mitochondrial membrane potential (MMP), expressed as Median Fluorescence Intensity (MFI) of Rhodamine 123.

**Figure 3:**
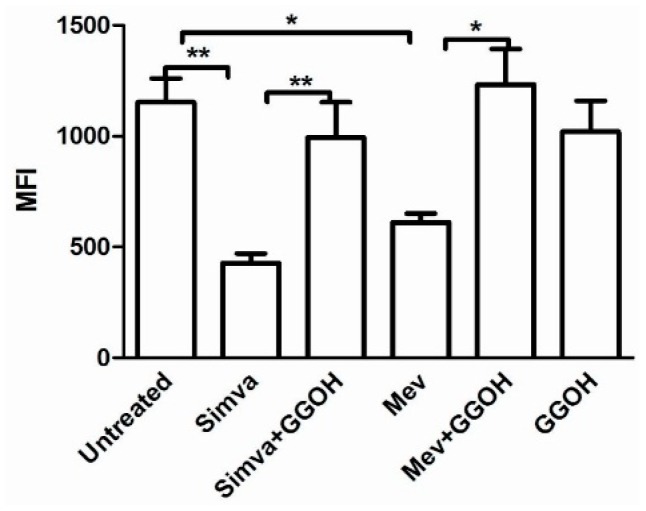
The Daoy cell line was incubated with Mevalonate (10 mM) and/or Simvastatin (10 μM) for 24 h and with GGOH (50μM) for an additional 24 h. Bars represent the Mean Fluorescent Intensity (MFI) of Rhodamine 123 ± SD of five independent experiments. Data analyses were performed with one-way ANOVA and Bonferroni post-test; * *p* < 0.05; ** *p* < 0.01.

In Figure 3, Simvastatin and Mevalonate strongly down regulated the MMP, resulting in an MFI of (427.7 ± 4) and (612.0 ± 40.4) respectively, compared to the control value (1153.0 ± 107.3).

Treatment with GGOH alone (1020.0 ± 139.7) did not significantly change MMP in comparison with the untreated condition, while in double treatment conditions GGOH upgraded the MMP in Simvastatin (994.5 ± 158.1) and Mevalonate (1233.0 ± 160.1), restoring the control MFI level. These results conclude that the statins and mevalonate in cells likely induce an alteration of ATP production related to an increase of MMP causing mitochondrial damage.

### 2.3. Effect of the Block of the Mevalote Pathway on Cell Viability

**Figure 4:**
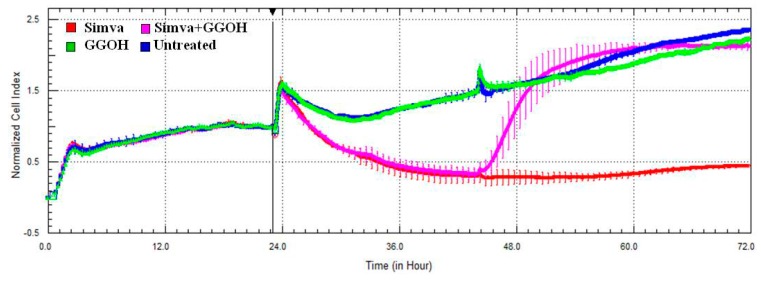
Impedance profiles after Simvastatin treatment. Daoy were seeded (5 × 10^5^/well) in E-plate and treated (at Time 0, after about 24 h seeding) with Simvastatin 10 µM and with GGOH 50 µM (after about 48 h seeding). The graph shows the Normalized Cell Index, at the time of the first stimulation (Time 0), indicated by the vertical black line. The experiment was performed in triplicate. Simva (red), GGOH (green), Simva + GGOH (pink), Untreated (blue).

The cytotoxic effect of Simvastatin on Daoy cells was confirmed with the xCELLigence system, that analyses in real-time the viability of cultured cells, through electrical impedance monitoring. The impedance graph (Figure 4) showed that Simvastatin (red line) induced a drastic decrease of the CI, which is an indicator directly proportional to the area covered by cells. This reduction is restored immediately after the administration of GGOH (pink line), added 24 h after the Simvastatin treatment. The GGOH treatment alone (green line) is comparable to the untreated condition (blue line) and does not perturb the growth curve of cells. In combination with the statin drug, however, it shows a “positive” effect on cell viability.

The results obtained from the Mevalonate treatment alone or in the presence of the isoprenoid are the same as those obtained by the statin, confirming the comparable kinetics of cell viability (data not shown).

### 2.4. Inflammasome Platform Expression in the Presence of a Block of the Mevalonate Pathway

**Figure 5:**
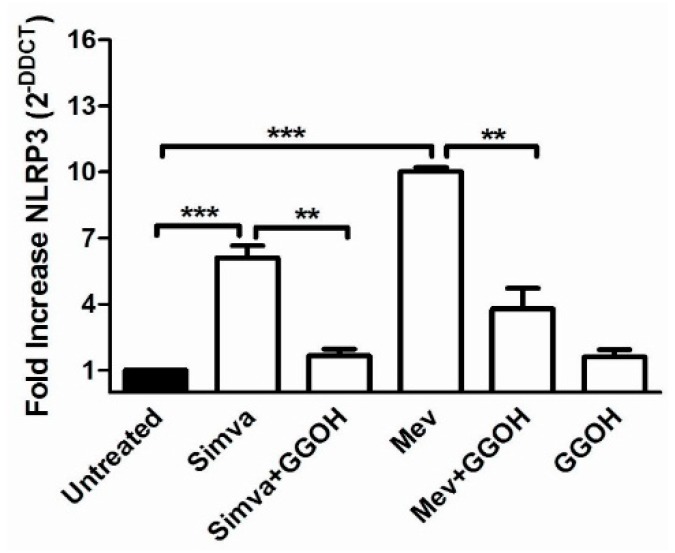
NLRP3 gene expression levels of Daoy cells after different drug treatments were calculated with respect to the untreated condition using the ΔΔCt method. All data represent the results of three independent experiments and are expressed as the mean of 2 ^−ΔΔCt^ ± standard error (SE). β-Actin was used for the normalization of all Ct values. Data analyses were performed with one-way ANOVA and Bonferroni post-test; ** *p* < 0.01, *** *p* < 0.001 versus untreated.

Inflammasome genes (*NLRP1, NLRP3, CASP1* and *ASC*) expression in the Daoy cell line after mevalonate and statin treatments, in presence or not of GGOH, was analysed and compared to unstimulated cells (Figure 5).

*NLRP3* expression was increased in Simvastatin (6.1 fold increase) and in Mevalonate treated-cells (10.1 fold increase) when compared with the untreated condition (normalized to 1).

A significant decrease in *NLRP3* mRNA expression was also measured after GGOH treatment on Simvastatin (1.6 fold) and Mevalonate (3.8 fold) (** *p* < 0.01, *** *p* < 0.001).

This isoprenoid proved its influence in reducing the inflammation caused by the block of the cholesterol traffic.

After 48 h of stimulation, neither the Simvastatin nor the Mevalonate treatments were able to modify the expression of the other inflammasome genes, *NLRP1*, *CASP1* and *ASC* (data not shown).

This results shows that among the inflammasomes, *NLRP3* is the most involved in the defense of the innate immune system in the Daoy cells.

### 2.5. Morphological Changes Correlated with Deregulation of the Mevalonate Pathway

**Figure 6:**
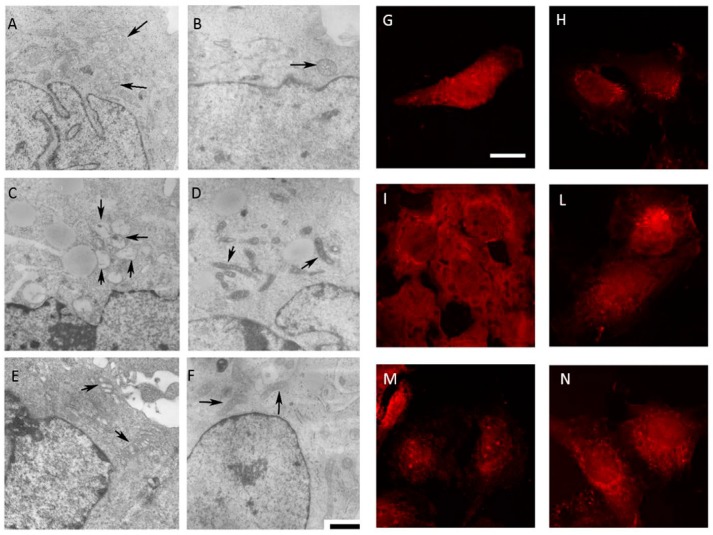
(1) Representative electron micrographs of Daoy cells incubated for 48 h in complete medium with Simvastatin (10 μM) and Mevalonate (10 mM) in the presence or absence of GGOH (50 μM) for 24 h. For each picture, an enlargement of the image is displayed, showing the presence of normal or damaged mitochondria (indicated by arrowheads). Scale bar, 1 μm; (2) Confocal microscopy of Daoy cells after different treatments stained with Mito-ID (red fluorescence). Many fields were examined and over 95% of the cells displayed the patterns of the respective representative cells shown here. Scale bar, 20 μm. (**A**,**G**) untreated condition; (**B**,**H**): GGOH treatment; (**C**,**I**): Simvastatin treatment; (**D**,**L**) Simvastatin + GGOH treatment; (**E**,**M**): Mevalonate treatment; (**F**,**N**): Mevalonate + GGOH treatment.

The Daoy cell line exhibits normal sized and oval-shaped mitochondria, with tubular cristae, with inner and outer membranes well defined (Figure 6A,G). The GGOH-treatment of the Daoy maintains the regular shape of cells in which the mitochondria present tubular cristae and the membranes can be observed in detail (Figure 6B,H). The biochemical block of the mevalonate pathway obtained with Mevalonate (Figure 6E,M) causes an evident misshape of damaged and swollen mitochondria, and several cristae are disrupted and leave large spaces in the inner area. Simvastatin-treatment leads to a similar situation of a shrunken mitochondria showing condensed cristae coalesced in small groups leaving empty spaces between them (Figure 6C,I).

The presence of GGOH in association with the block induced a significant change in morphology: the Mevalonate+GGOH treatment, indeed, produced amelioration in mitochondria morphology. They took a different shape with a thin and long form instead of the oval regular form (Figure 6F,N), and cristae are recognizable. They have a regular structure and distribution and the double membrane is better defined. The same results are observed in Simvastatin + GGOH cells treatment. GGOH co-treatment with Simvastatin or Mevalonate is able to reduce the injury of the mitochondria, enhancing their form and components (Figure 6D,L).

Further images with an enlargement of mitochondria can be found in the Supplementary material, to appreciate the different morphology after treatments (Supplementary Figure S2).

### 2.6. Supernatants Cytokines Levels in Daoy Cell Line Treated

The evaluation of cytokines production (IL-6 and TNF-α) was done 48 h after Simvastatin (10 μM) or Mevalonate (10 mM) treatments considering that this was the experimental time required to obtain the maximum increase in inflammatory markers. Simvastatin and Mevalonate induced a marked increase in IL-6 levels (4072 ± 449 and 9094 ± 358 respectively) in comparison to the untreated condition (2136 ± 105).

**Figure 7:**
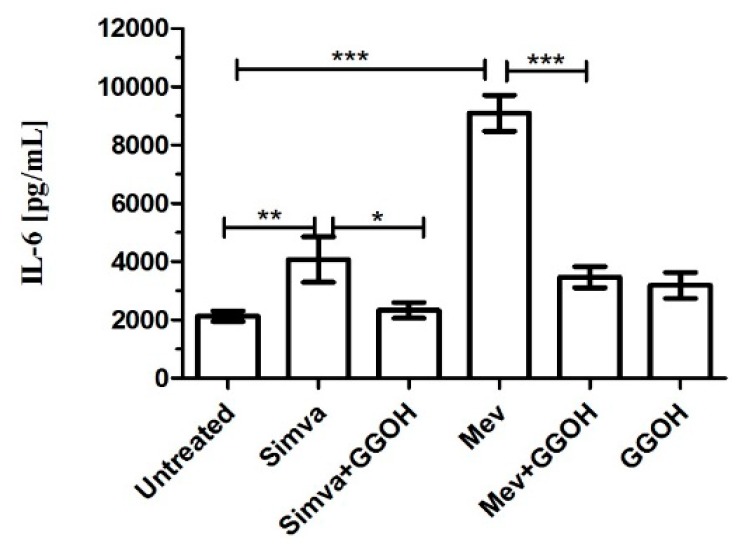
Deregulated IL-6 production after treatments on Daoy cells: treatments with Simvastatin (10 μM) and Mevalonate (10 mM) in the presence or absence of GGOH (50 μM) for 24 h. Geranylgeraniol was dispensed 24 h after the Simvastatin or Mevalonate treatments. The concentration values of IL-6 (pg/mL) were obtained from experiments performed in triplicate. IL-6 was significantly modulated with isoprenoids treatments. One way ANOVA test followed by the Bonferroni correction for multiple comparisons * *p* < 0.05, ** *p* < 0.01, *** *p* < 0.001.

The anti-inflammatory response to GGOH treatment (3192 ± 258) was studied in Simvastatin and Mevalonate conditions (2337 ± 157 and 3470 ± 213 respectively): the isoprenoid induced in both conditions a marked decrease in IL-6 production (Figure 7).

The level of TNF-α was under the lower limit of detection (LLOD) in all evaluated conditions, either because the level was very low or because this molecule is not produced by this neuronal line [21,22].

## 3. Discussion

The cholesterol metabolic pathway is a biochemical process highly regulated and associated with many life functions. The strong relationship between the regulation of this metabolic pathway and its implications on the inflammatory process are much studied as they are involved in the pathogenesis of many severe diseases, some of which are rare, and without a specific etiological treatment, as in the case of MKD [4]. Mandey *et al.* investigated the inflammatory response associated with an alteration of the mevalonate pathway in the MKD condition [16] in order to establish if the inflammatory response was associated with an accumulation or lack of intermediate compounds of the biochemical process. In our previous study, the results obtained from a monocytes cell line and from samples of MKD patients, showed that the shortage of isoprenoid end products rather than an excess of mevalonate is the cause of inflammatory events [23]. These data could justify the administration of exogenous isoprenoids (*i.e.*, GGOH) to compensate for the defective mechanism. Unfortunately, in the central nervous system this may not occur, because of the closed mechanism with which the biosynthesis and the consumption of cholesterol occur *in situ*. It is widely accepted that in the central nervous system, most cholesterol is produced in glia cells. On the contrary, neurons are responsible for the down-biosynthesis of cholesterol [24].

The role of microglia cells in the biosynthesis of neuroinflammation in MDK pathogenesis is very interesting, but it is difficult to find cell lines of human microglia. A recent pilot study conducted by our group with a co-culture of neuronal human cells and microglia mouse cells, showed that BV-2 is a murine microglia cellular model useful to have a biomarker of cytokines production [25]. Recently, however, a report showed that the knockdown of mevalonate kinase in BV-2 cells is not a suitable MKD model, because NALP3 expression and cell death does not correspond to the expression obtained from MKD patients [26].

Taking into account all these issues, it is crucial to establish if the neurons respond to the deregulation of the mevalonate pathway as it happen for monocytes, and if the inflammatory response is linked exclusively with the shortage of isoprenoids.

The block of the mevalonate pathway by Simvastatin or Mevalonate contribute both to the increase of the inflammatory markers and to the activation of the *NLRP3*-inflammosome. Moreover, these effects are associated with mitochondrial damage, as demonstrated by other authors in neurological disorders, due to impaired cholesterol metabolism [27,28,29,30].

The significant cell death in Simvastatin or Mevalonate-treated Daoy cells in comparison with the untreated condition shows that the block of the mevalonate pathway induces a dramatic morphological change in mitochondria, independently from the inhibitor. The mitochondrial unit is essential for energy production and redox homeostasis, responsible for cell life and death. Morphological changes are accompanied by alterations in the mitochondrial function, which can be related to the production of ROS (reactive oxidative species) and NO (nitric oxide), inducing a neuroinflammatory response leading to programmed cell death, which in this experimental setting was further confirmed by mitochondrial membrane potential.

Recent developments on the activation of NLRP3 and on damaged mitochondria, indicate that ROS produced by the dysfunction of mitochondria have a crucial role in priming NLRP3, even though the molecular mechanism underlying this process remains unclear [31,32].

Moreover, several cardiovascular disorders and metabolic syndromes, associated with cholesterol pathway deregulation, have been related to mitochondrial damage characterized by an abnormal balance of production between ROS and NO [33,34,35].

The capacity of isoprenoids, such as GGOH, to reverse these inflammatory conditions appears to be highly relevant. This capacity is supported by significant and evident changes in mitochondria morphology [36].

The capacity of GGOH to restore the pathway and reduce the induced inflammation has been highlighted through cytofluorimetric analysis and gene expression. Based on recently published data we known that cell death associated to the block of the mevalonate pathway follows the mitochondrial pathway, which is caspase-9 and caspase-3 dependent [14,15]. Observing these data, we can hypothesise that isoprenoids (such as GGOH) are able to restore the physiological cellular homeostasis, influencing the main actors of the mitochondrial damage (such as the components of the Bcl-2 family members, Bax and Bak).

Moreover, IL-6, commonly considered as an inflammatory marker, can be adopted as an additional indicator of the effect of GGOH: the isoprenoid is able to reduce the inflammatory marker in both conditions by which we blocked the pathway. The evaluation of the TNF-α production has not led, however, to significant results, and we believe the rationale behind this result should be searched in the kinetics synthesis of the pro-inflammatory cytokines after a stimulus [37,38,39].

In conclusion, despite the important results obtained from this model, the mechanism by which the blockage of the biochemical process induces neuroinflammation is poorly understood.

It would be easier to study this mechanism by working with neuronal cell lines from MKD patients, but the main problem researchers are facing is represented by the difficulty of collecting biological material and data. Indeed, the only information they can collect derives from the clinical observation of patients showing psycho-motor disorders or from post-mortem analyses. For ethical reasons, it is not possible to design experiments to directly study the neuronal tissue of patients, since this type of analysis would also require samples collected from healthy age-matched controls.

The adoption of an MKD-neuronal biochemical model leads us to consider these results as preliminary, but as a good starting point to better understand the link between the neurological involvement and inflammatory aspects. The acquired knowledge on the neuronal apoptotic mechanism could allow researchers to develop novel drugs for the still orphan MKD deficiency.

## 4. Materials and Methods

### 4.1. Reagents and Cell Culture

Geranylgeraniol (GGOH), Simvastatin (Simva, Mevinolin from *Aspergillus* *terreus*), and Mevalonate (Mev) were obtained from Sigma Chemical Co. Aldrich (St. Louis, MO, USA) and dissolved in saline solution (Simvastatin) or ethanol (Sigma Chemical Co. Aldrich, St. Louis, MO, USA), which did not exceed 0.01% final concentration per well.

Daoy (human derived desmoplastic cerebellar medulloblastoma cell line), obtained from ATCC, were cultured in EMEM (Eagle’s Minimum Essential Medium) (Euroclone, Italy), supplemented with 10% foetal bovine serum (FBS, Euroclone, Italy), 2 mM glutamine and penicillin streptomycin anphotericin B 1× solution (Sigma Chemical Co. Aldrich, St. Louis, MO, USA).

Twenty-four hours after seeding in a 6 wells plate, 2 × 10^5^ cells were stimulated with Mev (10 mM) or Simva (10 μM). After 24 h from the first treatment, Daoy were treated also with GGOH (50 μM). Cells were incubated for further 24 h and were then harvested for the analysis.

### 4.2. Flow-Cytometry Analysis: Programmed Cell Death Assays and Mitochondrial Membrane Potential Assay

The programmed cell death of Daoy was analysed by flow cytometry using Annexin V (A) and Propidium Iodide (PI) staining. Cells were stained with FITC-conjugated Annexin V and Propidium Iodide (Annexin V-FITC Apoptosis Detection Kit, Immunostep, Spain) following the customer indications. Briefly, 24 h after last stimulation, cells were harvested from the plate with a 0.5% Trypsin-EDTA solution (Sigma Chemical Co. Aldrich St. Louis, MO, USA) and washed with PBS. 5 × 10^5^ cells were resuspended in the manufacturer’s Binding Buffer, stained with A and PI for 15 min, then acquired on flow cytometer. To evaluate the percentage of death cells, debris was excluded from the plot based on the scatter, while the apoptotic and necrotic cells were characterized on the basis of emitted fluorescence.

Changes in mitochondrial membrane potential (Δ*Ψ*m) were determined by Rhodamine 123 staining. In brief, after treatments, cells were harvested from the plate, washed in PBS and suspended in 500 μL PBS. Cells were then stained with 10 μg/mL Rhodamine 123, at 37 °C for 30 min, under agitation. Rhodamine 123 median fluorescence intensity (MFI) was measured by flow cytometry.

For all the experiments, data were acquired with CyAn ADP analyser and Summit software (Beckman Coulter, CA, USA), then analysed with FlowJo software (version 7.6, Treestar, Inc. Ashland, OR, USA).

### 4.3. The xCELLigence System and Analysis of Impedance

The effect of the pharmacological treatment (Simvastatin, Mevalonate and GGOH) was assessed with the xCELLigence RTCA DP Instrument (Roche, Penzberg, Germany), that analyses the electrical impedance due to cell adhesion on well bottom, covered by gold microelectrodes. With this automatic system, the number, the morphology and the viability of attached cells are displayed as an alteration of the impedance, that is recorded in real time as an adimensional parameter, called “Cell Index” (CI). A decrease in CI, after adding a pharmacological compound, could be due to a detachment or to the death of cultured cells. Briefly, for this analysis, 5 × 10^3^ Daoy were seeded in a 16 well plate (E-plate) in 200 μL of complete medium, and cultured in 5% CO_2_ at 37 °C. The cells were stimulated following the same experimental design described in Section 4.1. The impedance was measured every 15 min before the pharmacological treatment and every 2 min after drug administration. This assay was repeated twice in triplicate.

### 4.4. RNA Isolation and Real Time-PCR

Total RNA was isolated using the RNAqueous-Micro kit (Ambion, Thermo Fisher Scientific, Waltham, MA, USA). First-strand complementary cDNA was synthesized from 1 μg of extracted RNA in a 20 μL reaction volume using High Capacity cDNA Reverse Transcription Kits (Applied Biosystems, Thermo Fisher Scientific, Waltham, MA, USA) according to the manufacturer’s protocol.

*NLRP1, NLRP3, CASP1* and *PYCARD* were amplified with specific TaqMan^®^ Gene Expression Assays (Hs00248187_m1, Hs00366465_m1, Hs00354836_m1, hs00203118_m1) with TaqMan Gene Expression Master Mix (Applied Biosystems, Thermo Fisher Scientific, Waltham, MA, USA) using the 7500 Fast DX Real Time PCR Instrument (Applied Biosystem, Thermo Fisher Scientific, Waltham, MA, USA). The housekeeping gene *ACTB (*Hs99999903_m1) was used for normalization. Raw fluorescent data of Ct values were corrected based on PCR efficiencies using LinRegPCR [40]. The relative quantitative expression of the selected transcript was calculated using the ΔΔCT methods [41]. Experiments were performed in triplicates and analysis of each experiment was executed in duplicate wells, in the same experimental setup described in Section 4.1.

### 4.5. Immunohistochemistry

Pellets were fixed with 2.5% glutaraldehyde (Electron Microscopy Sciences, Hatfield, PA, USA) in 0.1 cacodylate buffer, pH 7.3, for 1 h at room temperature, rinsed twice for 10 min in 0.1 cacodylate buffer and postfixed with 1% osmium tetroxide in the same buffer for 1 h at 4 °C (Hayat, Basic techniques for transmission electron microscopy, Academic Press, Inc., Orlando, FL, USA, 1986). Pellets were dehydrated in ascending alcohols, treated with propylene oxide and embedded in Araldite (Electron Microscopy Sciences, Hatfield, PA, USA). Ultrathin sections of the samples were cut on a Top Ultra 150 ultramicrotome (Pabish, Germany) and collected on 300-mesh copper grids. The grids were stained with uranyl acetate and lead citrate and examined at 80 kV using a Jeol JEM 100S transmission electron microscopy.

### 4.6. Confocal Microscopy

Daoy cell line was primed with GGOH on coverslips (4 × 10^5^ cells per coverslip), placed in a 12 well plate and treated for 24 h with Mevalonate or Simvastatin. Cells adhered on coverslips, were then washed with PBS and the intracellular localization of fullerenes was traced using the mitochondrial marker MitoTracker Red (CMXRos) (Molecular Probes, Eugene, OR, USA) for 15 min at 37 °C following the instructions of the manufacturer. Cells were examined using a Nikon C1-SI confocal microscope (TE-2000U) equipped with a 60× oil immersion lens.

MitoTracker Red was visualized using the 561 nm length diode laser for excitation and a 590 dichroic mirror with a 50 nm band emission filter was used to record emission. All images were acquired in the linear intensity window and with no visible saturation points. The pixel resolution was 100 nm for X and Y axis and z-step was established at 300 nm. Representative images are z-projection performed using standard deviation algorithm in ImageJ software (NIH).

### 4.7. Determination of Cytokines Release

The analyses of cytokines (including: interleukin 6 (IL-6), and tumour necrosis factor α (TNF-α), were performed on supernatant samples using a magnetic bead-based multiplex immunoassays (Bio-Plex^®^) (BIO-RAD Laboratories, Milano, Italy) following manufacturer’s instructions. Data from the reactions were acquired using the Bio-Plex^®^ 200 reader, while a digital processor managed data output and the Bio-Plex Manager^®^ software returned data as Median Fluorescence Intensity (MFI) and concentration (pg/mL).

### 4.8. Data Analysis

All results are expressed as the mean ± standard deviation (SD). Statistical significance was calculated using a one-way analysis of variance (ANOVA) and Bonferroni post-test correction in the case of multiple comparisons. Analysis was performed using GraphPad Prism software (version 5.0).

## Supplementary Materials

Supplementary materials can be found at http://www.mdpi.com/1422-0067/17/3/365/s1.
