# Supplementary material for: Geranylgeraniol and Neurological Impairment: Involvement of Apoptosis and Mitochondrial Morphology
Source: Int J Mol Sci. 2016 Mar 11;17(3):365. doi: 10.3390/ijms17030365 (PMC4813225; doi:10.3390/ijms17030365)
Supplement: Supplementary file 1 [file ijms-17-00365-s001.pdf]

Supplementary Materials: Geranylgeraniol and Neurological Impairment: Involvement of Apoptosis and Mitochondrial Morphology

Annalisa Marcuzzi, Elisa Piscianz, Marina Zweyer, Roberta Bortul, Claudia Loganes, Martina Girardelli, Gabriele Baj, Lorenzo Monasta and Claudio Celeghini

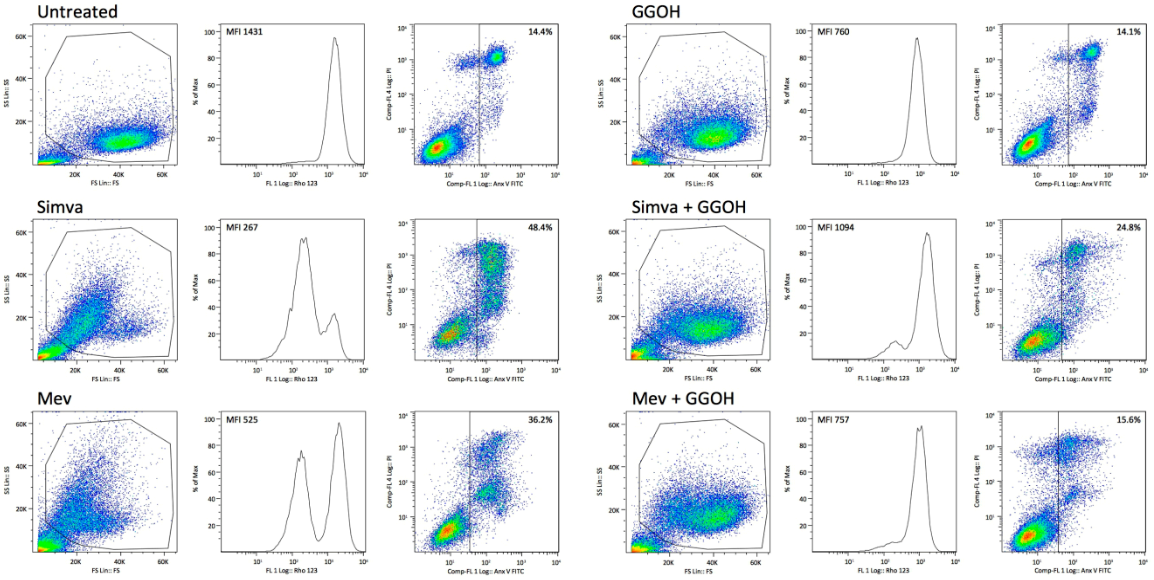

Figure S1. Rappresentative flow cytometry graphs of Daoy cells treated with 48 h in complete medium with Simvastatin (10µM) and Mevalonate (10 mM) in the presence or absence of GGOH (50 µM) for 24 h. The graphs show the Mean Fluorescent Intensity (MFI) of Rhodamine 123 and the percentage of Annexin V positive cells after the treatments.

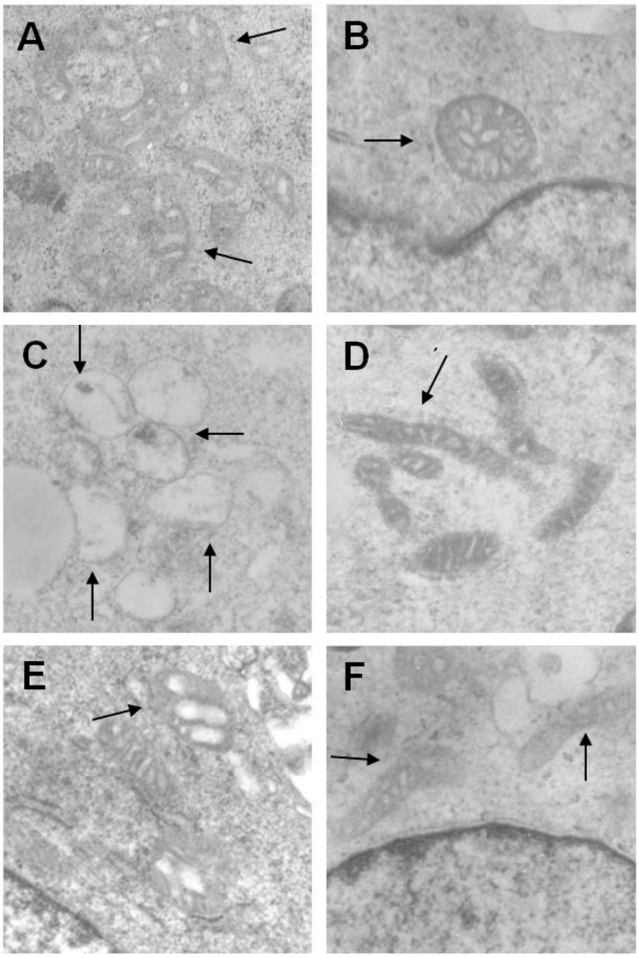

Figure S2. Representative electron micrographs of Daoy cells incubated for 48 h in complete medium with Simvastatin (10 μM) and Mevalonate (10 mM) in the presence or absence of GGOH (50 μM) for 24 h. Each picture show an enlargement of normal or damaged mitochondria (indicated by arrow). (**A**) untreated condition; (**B**) GGOH treatment; (**C**) Simvastatin treatment; (**D**) Simvastatin + GGOH treatment; (**E**) Mevalonate treatment; (**F**) Mevalonate + GGOH treatment.
